# Displaced Supracondylar Humerus Fractures in Children – Are They All Identical?

**DOI:** 10.5704/MOJ.1707.017

**Published:** 2017-07

**Authors:** SK Gera, MCH Tan, YG Lim, KBL Lim

**Affiliations:** Department of Orthopaedics, KK Women's and Children's Hospital, Singapore

**Keywords:** humerus supracondylar fracture, elbow fracture, paediatric, older child, adolescent

## Abstract

**Introduction:** This study aims to ascertain if there are any differences in supracondylar fractures between children under seven years of age and those above 7 years of age.

**Materials and Methods:** All cases of displaced humerus supracondylar fractures that required surgical stabilization were identified and retrospectively reviewed. Demographic data, mode of injury, associated neurovascular injuries and details of surgery performed were obtained from clinical records. The Gartland classification and the extent of comminution of fractures were also documented from review of radiographs.

**Results:** One hundred and twelve children were included in this study, of whom 61 (54.46%) were younger than seven years of age while 51 (45.5%) were aged seven years or older. Children aged seven or older had a greater incidence of associated neurological deficit at presentation (p=0.046). Of the six patients with nerve injury in the older age group, one patient (16.7%) had a radial nerve injury, two patients (33.3%) had ulnar nerve injuries while another two patients (33.3%) had median nerve injuries. There was one patient (16.7%) with both median and ulnar nerve injuries. Comminuted fractures were also more common in the older children (p=0.004). No significant differences were demonstrated between the groups with regard to age, gender and mechanism of injury, laterality, incidence of open fracture, vascular injuries and operative time.

**Conclusion:** Children aged seven years or older who sustain supracondylar humeral fractures tend to get more comminuted fractures. There is also a higher incidence of associated neurological injury. These cases must be carefully examined for at presentation and parents need to be appropriately counselled about them.

## Introduction

Supracondylar humerus fractures (SCHFs) account for 18% of all paediatric fractures and up to 60% of paediatric elbow fractures^1^. Studies have shown that these fractures occur commonly in children aged between 5-10 years and up to 70% of patients sustain the injury after a fall on the outstretched hand^3^. While associated neurovascular injury is uncommon, it can occur in higher energy injuries and should be carefully evaluated.

Undisplaced or partially displaced SCHFs can be treated non-operatively by cast immobilization. Rotationally unstable (Gartland IIB) fractures and completely displaced fractures require surgical fixation, usually with closed reduction and percutaneous pinning (CRPP).

In general, older children tend to sustain more comminuted SCHFs, with 2-part fractures seen less often. Neurological injuries and open fractures were also noted to be more common at presentation in older children^4^. As a result of these factors, closed reduction seemed more challenging, needing open reduction at times^5^ and surgical time was longer than two-part fractures that were more commonly seen in younger children.

This observation provided the impetus to conduct this retrospective cohort study to identify differences in fracture pattern in children of different age groups. Despite a large number of studies on SCHFs, there is a dearth of publications comparing SCHFs in different age groups within the paediatric population.

## Materials and Methods

This is an IRB approved study conducted at a tertiary children’s hospital. A total of 112 cases of SCHFs requiring surgical stabilization were identified between 2009 and 2013. This was a retrospective analysis of patient records and radiographs. Demographic data, mechanism of injury like fall on outstretched hand (FOOSH), fall on flexed elbow, side of injury, presence of open fracture, grade of fracture, flexion versus extension type of fracture, displacement of fracture, presence of medial spike, medial column comminution, fracture comminution, presence of neurovascular injury, associated injuries, operating time, closed versus open reduction, and surgeon level were recorded and analyzed. These details were obtained from both clinical notes and electronic medical records.

The Gartland classification and the extent of comminution were documented from review of radiographs. Details regarding operating time, surgeon level and type of reduction were obtained from operation notes and radiographs.

Patients identified were divided into two age groups: younger than seven, and seven years or older. Linear regression, logistic regression and Pearson’s Chi-Square tests were run on SPSS version 17.0 in data analysis.

## Results

A total of 112 cases were included in this study. Of these, 61 (54.46%) children were younger than seven years while 51 (45.54%) were aged seven or older, with a mean age of 6.82 (range: 1-14). There were 75 males and 37 females.

**Fig. 1:**
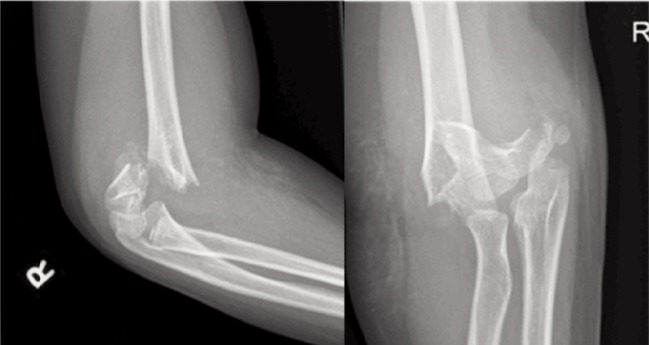
Supracondylar fracture with comminution in an older child aged >7 years.

**Fig. 2:**
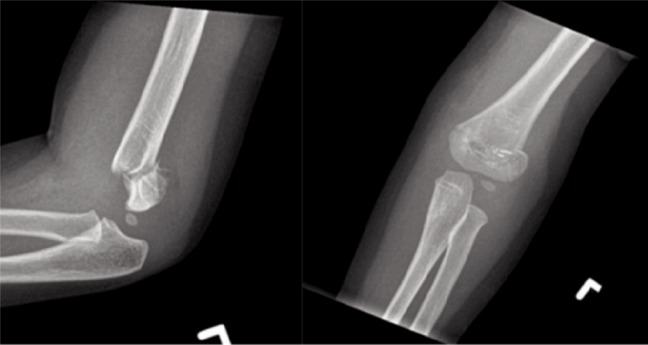
Supracondylar fracture with no comminution in a child aged <7 years.

In this study, we found a correlation between the presence of comminution and age: older children had a higher incidence of comminuted fracture (p= 0.002). Data on comminution was not available for one child; however, of the remaining 111 children, 46 (41.1%) sustained comminuted fractures (Fig. 1). Twenty-nine of 50 children (56.9%) in the older age group had comminuted fracture as compared to 17 of 61 (27.9%), in the younger age group (Fig. 2); this difference was statistically significant.

In this series, children in the older age group had a higher incidence of nerve injury (p=0.046). Overall, seven (6.3%) children were found to have nerve injury at initial presentation. One (1.6%, n=61) was from the younger age group while six of 51 (11.8%) were from the older age group. Of the six children with nerve injury in the older age group, one patient (16.7%) had sustained a radial nerve injury, two (33.3%) ulnar nerve injury and two (33.3%) median nerve injury. There was one child (16.7%) who had sustained both median and ulnar nerve injuries. The only child in the younger age group had sustained a median nerve injury.

Table I summarizes the rest of the results in this study. No statistically significant differences between the groups were found for the following factors, such as gender (p=0.069), mechanism of injury (p=1), laterality (p=0.69), Gartland classification (p=1), flexion versus extension type (p=0.512), direction of displacement (p=0.634), medial spike (p=0.902), medial column comminution (p=0.32),vascular injury (p=0.119), associated injuries (p=0.108), type of reduction (open versus closed) (p=0.272) and the level of surgeon (p= 0.219).

**Table I:** Summary of results

		**Younger than 7 years (n=61)**	**7 years or older (n=51)**
Sex (p=0.069)	Male	35 (57.38%)	38(74.51%)
	Female	26(42.625%)	13(25.49%)
Mechanism of injury(p=0.843)	FOOSH	25(43.10%)	23(45.10%
	Flexed elbow	33(56.9%)	28(54.90%)
Laterality (p=0.600)	Right	21(34.43%)	20(39.22%)
	Left	40(65.57%)	31(60.78%)
Garland classification(p=0.855)	2b	2(3.28%)	2(3.28%)
	3	59(96.72%)	49(96.08%)
Type of fracture (p=0.512)	Flexion	2(3.28%)	3(5.88%)
	Extension	58(96.72%)	48(94.12%)
Displacement (p=0.634)	Posteromedial	29(50.00%)	25(52.08%)
	Poster lateral	20((34.48%)	14(29.17%)
	Posterior	8(13.79%)	6(12.5%)
	Others	1(1.73%)	3(3.25%)
Medial spike (p=0.885)	Yes	10(16.95%)	9(18.00%)
	No	51(83.6%)	42(82.4%)
Comminution*(p=0.002)	Yes	17(27.9%)	29(58.00%)
	No	44(72.1%)	22(41.2%)
Nerve injury*(p=0.046%)	Yes	1(1.6%)	6(11.8%)
	No	60(98.4%)	45(88.2%)
Vascular injury (p=0.108)	Yes	0(0%)	2(3.2%)
	No	61(100%)	49(96.72%)
Associated injuries (p=0.108)	Yes	3(4.92%)	0(0%)
	No	58(95.08%)	51(100%)
Type of reduction (p=0.272)	Open	0(0%)	1(1.96%)
	Closed	61(100%)	50(98.04%)
Surgeon level (p=0.129)	Registrar	31(50.82%)	20(39.22%)
	Consultant	30(49.2%)	31(60.8%)
Operating time (p=0.085)	Mean time	20.36	23.63

All 112 children had sustained closed fractures. Despite a higher incidence of comminution and associated nerve injury in the older group, there was no statistically significant correlation between operating time and age group on Mann-Whitney testing (p=0.505). The mean operating time for the younger age group was 20 minutes (range: 7-60 minutes) while the mean operating time was 24 minutes (range: 6-100 minutes) for the older age group.

## Discussion

Our results suggest that SCHFs in children aged seven years or older are more likely to be associated with neurological injury than those in younger children. These fractures are also more likely to be communitted, rendering them potentially more unstable than a two-part fracture.

Compared with younger children, children aged seven years or older are larger in stature, heavier and more likely to participate in strenuous activities and sports that often involve greater heights and speeds. For example, older children would not just cycle or rollerblade but would attempt stunts at high speed. As such, older children may sustain higher energy injuries compared with those sustained by their younger counterparts. Fracture fragment excursion at the time of impact is probably greater in the older children, resulting in nerve injury and significant soft tissue swelling. Neurological complications resulting from SCHFs are well recognized and reported^6,7^. The overall incidence of neurological injury of 6.3% in this series falls within the range of 5-19% as previously reported^8^.

Bone composition changes with growth. It is well established that the bone of an older child has less collagen, thinner periosteal sleeve^4^ and cancellous bone compared with a younger child. The implications are that older children will fracture more readily, and their fractures are more likely to be comminuted rather than a simple two-part fracture.

Surgical fixation with closed reduction and percutaneous pinning of the more comminuted fractures in older children may prove more challenging given their greater instability. While satisfactory closed reduction can be achieved, it might require multiple pins to maintain stability of the reduced fracture^4^. However the larger and more prominent bony landmarks in the older child may facilitate the identification of entry points for the percutaneous pins. Indeed, operative time for both age groups was not statistically significant in this study, despite the greater comminution seen in the older age group. The larger metaphyseal bony contact area at the fracture site may also offer some stability in the older child.

## Conclusion

Supracondylar humeral fractures in children aged seven years or older are more likely to be comminuted and associated with neurological injury. Changes in bone composition and higher energy injuries in older children may at least in part explain these findings. A meticulous clinical examination at presentation is imperative so that associated neurological and other injuries are not missed.

The findings in this study will add to the literature on supracondylar humeral fractures in children and will also have some bearing on the management of these fractures in children aged seven years or older. Anatomical closed reduction of these comminuted fractures may not be straightforward as reducing a two-part supracondylar humerus fracture in the younger child. However, larger, more prominent bony landmarks around the elbow make the identification of pin entry points simpler.
